# Microbial Quality of Treated Wastewater and Borehole Water Used for Irrigation in a Semi-Arid Area

**DOI:** 10.3390/ijerph18168861

**Published:** 2021-08-23

**Authors:** Pholosho Mmmateko Kgopa, Phatu William Mashela, Alen Manyevere

**Affiliations:** 1 Department of Plant Production, Soil Science and Agricultural Engineering, University of Limpopo, Private Bag X 1106, Sovenga 0727, South Africa; phatu.mashela@ul.ac.za; 2 Department of Agronomy, University of Fort Hare, Private Bag X1314, King William’s Town Rd, Alice 5700, South Africa; AManyevere@ufh.ac.za

**Keywords:** microbial contamination, spatiotemporal effects, treated wastewater quality, water re-use

## Abstract

The current study investigated the distribution of microbial populations and diversity in treated wastewater used for irrigation at the University of Limpopo Experimental Farm (ULEF), from different stages of post treatment disposal at Mankweng Wastewater Treatment Plant (MWTP) in Limpopo Province, South Africa. The study was arranged in a 4 × 5 factorial experiment, which studied the interactive effects of four collection points and five months of sampling, with borehole water used as a reference point. Water samples were analyzed for bacteria, helminths, and protozoa. All data were transformed and subjected to factorial analysis of variance. The site–time interactions were significant for *Salmonella* spp. and *Ascaris lumbricoides*, whereas collection point was significant for all variables. In conclusion, movement and storage of water post treatment at MWTP were able to improve the microbial quality of the treated wastewater disposed for irrigation at ULEF.

## 1. Introduction

Irrigated agriculture in South Africa plays a major role in food security, job creation, and wealth generation [1]. However, water scarcity is a major challenge that affects agriculture as a whole, including food production through irrigation [2]. Water scarcity, which can be described as the imbalance between the availability and demand [3], results in food scarcity, since 70% potable water is allocated to agriculture. As a result, agriculture could be both the cause and the victim of water scarcity. Since the introduction of sedentary agriculture, water scarcity has become a major challenge in various production systems [2]. Worldwide, 1.1 billion people lack access to water, and a total of 2.7 billion experience water scarcity for at least a month in a year, with predictions that the situation will only worsen with time as climate change intensifies [4]. A number of factors including drought spells, dry climates, and overpopulation in different countries have been reported to accelerate water scarcity problems [5]. As agriculture is the main user of fresh water, irrigation becomes negatively affected, leading to less production of food [6]. In Tunisia, predictions suggest that agricultural activities would be negatively affected due to the decrease in availability of irrigation water due to increasing challenges due to water scarcity [7]. Furthermore, Khan [2], reported in their study conducted in Pakistan that water scarcity had a negative impact on crop production and the livelihood of smallholder farmers.

Limpopo Province in South Africa was, between 2012 and 2016, among the provinces declared drought-stricken [8]. As such, some farmers thereafter resorted to using treated wastewater for irrigation. The University of Limpopo Experimental Farm (ULEF) (23°50′42.86′′ E; 29°42′44′′ S) has also adopted the strategy for irrigation of agricultural crops including leaf, root, and bulb vegetables. However, studies advise that all treated municipal wastewater should be viewed as potential carriers of pathogens with the potential ability to serve as a source of contamination of the agricultural produce [9,10].

Standards for safe wastewater use have mainly been developed to avoid risks associated with soil, plant, and human health [11]. The use of treated wastewater is associated with health risks due to the possibility of the presence of pathogens leading to contamination of produce [12]. As such, contaminated water has a potential of creating and spreading diseases through contaminated soils and agricultural produce [9]. A number of studies have shown the relationship between treated wastewater use and food-borne illnesses like cholera, gastroenteritis, and anemia [13,14]. The World Health Organization also reported on estimated deaths due to food-borne diseases in different countries [15]; with some cases resulting from irrigation with treated wastewater. Therefore, this study investigated the distribution of microbial populations and diversity in treated wastewater used for irrigation at ULEF at different stages of post treatment disposal at Mankweng Wastewater Treatment Plant (MWTP). The objective of this study, therefore, was to quantify and compare the spatial and temporal chemical and biological quality of the treated wastewater from the treatment plant release point, Pond-16 exit, through the night-dam entry and exit points at the ULEF, with the portable borehole water serving as a standard.

## 2. Materials and Methods

### 2.1. Study Site Description, Design and Treatments

**Figure 1:**
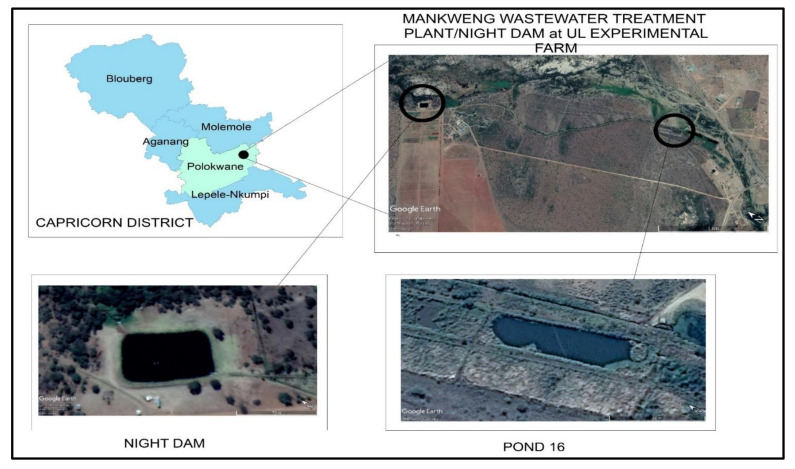
Study site, a pathway from pond 16 of MWTP to ULEF night dam along a 2.9 km canal.

**Figure 2:**
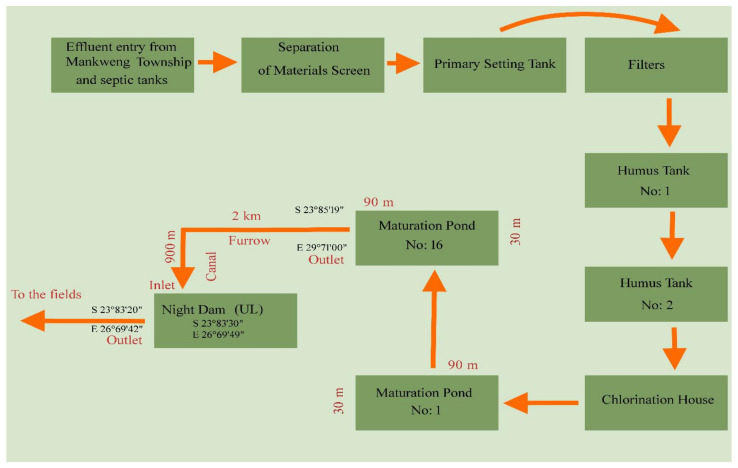
Schematic representation of the sampling points from the Mankweng Wastewater Treatment Plant in Mankweng and the receiving dam at UL Experimental Farm.

The study was conducted at various points of Mankweng Wastewater Treatment Plant (MWTP) (23°51′23.61′′ S; 29°42′27.24′′ E) and the University of Limpopo Experimental Farm (ULEF) (23°50′42.86′′ S; 29°42′44.35′′ E) (Figure 1). The study comprised two factors, namely, four sampling points (pond 16, night dam entry, night dam exit and borehole) as summarized in Figure 2, and five sampling months. The MWTP received effluent from a number of industries in Mankweng Township (23°87′84′′ S, 29°71′37′′ E), namely, the University of Limpopo (23°88′71′′ S, 29°73′84′′ E), Mankweng hospital (23°87′90′′ S, 29°72′61′′ E), two local shopping centers, filling stations, various human settlements, and runoff water from buildings. The effluent undergoes physical, biological, and chemical (chlorine) treatments prior to disposal into the furrow for conveying treated wastewater to the night-dam at ULEF (Figure 2). Water samples were collected on the 15th of each month from July 2019 to November 2019 to represent the monthly range from colder to cooler and then warmer months.

### 2.2. Water Sampling and Isolation of Bacteria

Once a month, water samples were collected in 500 mL sterile glass bottles and transported on ice to UL Water and Sanitation laboratory for analysis. Sample dilutions at 10^5^ prepared from three volumes of 100 mL were filtered through a 0.45 μm Whatmann micro-filter using a water filtering manifold system [16]. The membranes were aseptically placed up on plates with appropriate selective media ensuring that no air bubbles were trapped [17]. The selective media used were as follows: XLD agar used as a selective medium for *Salmonella* spp. and *Shigella* spp., Membrane-Thermotolerant *Escherichia coli* Agar (mTEC) for *E. coli*, Thiosulfate-Citrate-Bile Salts-Sucrose (TCBS) for isolation of *Vibrio fluvialis*, *Vibrio parahaemolyticus*, *Vibrio cholera*, and *Vibrio alginolyticus*. m-FC agar was used for isolation of Fecal coliform.

### 2.3. Water Sampling and Detection of Helminths/Protozoa

Once a month, water samples were collected in sterile 5 L bottles in three replicates, transported on ice to the Water Microbiology Laboratory, Council for Scientific and Industrial Research (CSIR), Pretoria, South Africa, for detection of *Entamoeba histolytica*, *Schisostoma mansoni*, and *Ascaris lumbricoides*. The detection was done following the Bailenger (WHO) method, which involved detection and identification of eggs in water samples using a microscope [18].

### 2.4. Statistical Analysis

All microbial data were transformed using log_10_(x + 1) to homogenize the variances [19], and subjected to factorial analysis of variance (ANOVA) using Stata 12 software [20]. Interactive effects of sampling site and sampling time were further assessed using the two-way matrix tables [19]. Treatment means were separated using Duncan multiple range and Fisher’s least significant test (*p* ≤ 0.05) and presented in terms of relative impact (RI%), which was computed as follows using the relation:

*R.I.* (%) = [(*Treated wastewater/Borehole*) − 1] × 100.

The mean microbe variables of samples collected at the borehole site were used as reference points. The mean helminths and protozoa of samples collected from Pond 16 were used as a reference point. Unless otherwise stated, treatment effects were described at the 5% level of probability (*p* < 0.05).

## 3. Results

### Bacteria, Helminths, and Protozoa Counts as Affected by Site and Time

**Figure 3:**
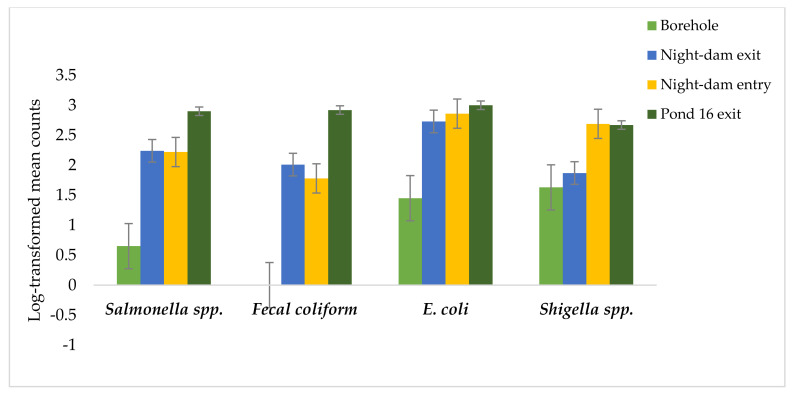
Log-transformed mean counts of *Salmonella* spp., *Shigella* spp., *Escherichia coli*, and fecal coliform in treated wastewater relative to that from borehole water used for irrigation of various crops.

The site–time interaction was significant on *Salmonella* spp., but had no significant effects on *Shigella* spp., *E. coli*, or fecal coliform. Collection site was highly significant (*p* ≤ 0.01) for *Shigella* spp., *E. coli*, and fecal coliform, whereas the sampling time had no significant effects on any of the three variables. Relative to borehole water, night-dam exit, night-dam entry, and Pond 16 increased *Salmonella* spp. 243, 239, and 343%, respectively (Table 1, Figure 3). Fecal coliform was not detected in borehole water (Figure 3). Relative to borehole water, the night-dam exit, night-dam entry, and Pond 16 increased *E. coli* by 88, 97, and 106%, respectively. Relative to borehole water, the night-dam exit, night-dam entry, and Pond 16 increased *Shigella* spp. by 15, 65, and 64%, respectively (Table 1, Figure 3).

**Figure 4:**
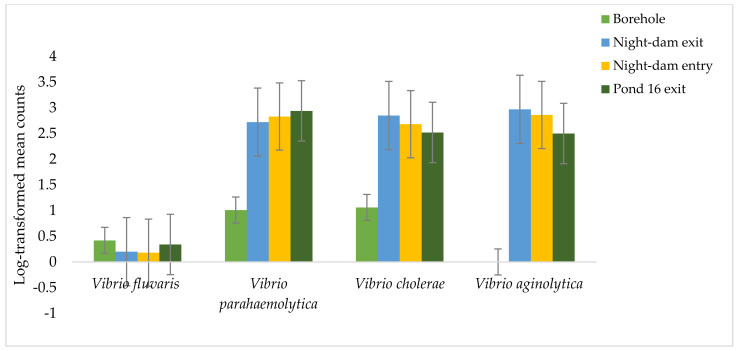
Log-transformed mean counts of *Vibrio fluvaris*, *Vibrio parahaemolytica*, *Vibrio cholerae*, and *Vibrio aginolytica* as affected by collection site along the wastewater treatment pathway from Pond 16 exit to night-dam exit relative to those in the borehole water.

The site–time interaction and the sampling time were not significant for any combination of variables. However, the collection site had highly significant effects on the four *Vibrio* species, *V. fluvaris*, *V. parahaemolytica*, *V. cholera*, and *V. aginolytica*. Relative to the borehole, the night-dam exit, night-dam entry, and Pond 16 decreased *V. fluvaris* by 51, 58, and 19%, respectively. Relative to borehole, the night-dam exit, night-dam entry, and Pond 16 increased *V. parahaemolytica* by 169, 180, and 191%, respectively. Relative to borehole, the night-dam exit, night-dam entry, and Pond 16 increased *V. cholera* by 169, 153, and 138%, respectively. There was no presence of *V. aginolytica* in the borehole water (Table 2, Figure 4).

**Figure 5:**
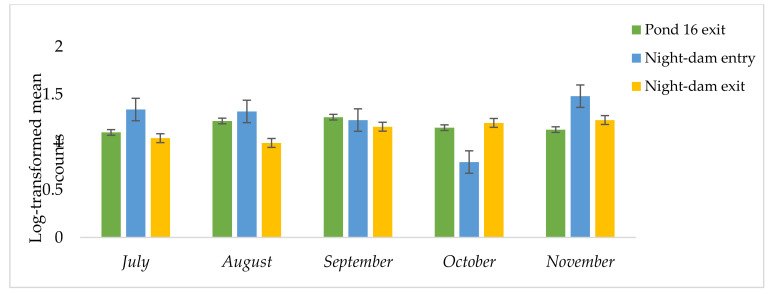
Log transformed *Ascaris lumbricoides* counts as distributed along the wastewater treatment pathway from Pond 16 exit to night-dam exit for five months in 2019.

The site–time interaction was significant for *A. lumbricoides*, but was not significant for *S. mansoni* or *E. histolytica*. Collection site was highly significant for *S. mansoni* and *E. histolytica*. Sampling time was not significant for *S. mansoni* or *E. histolytica.* Relative to July, Pond 16 increased *A. lumbricoides* by 11, 14, 4, and 3%, in August, September, October, and November, respectively (Table 3, Figure 5).

**Figure 6:**
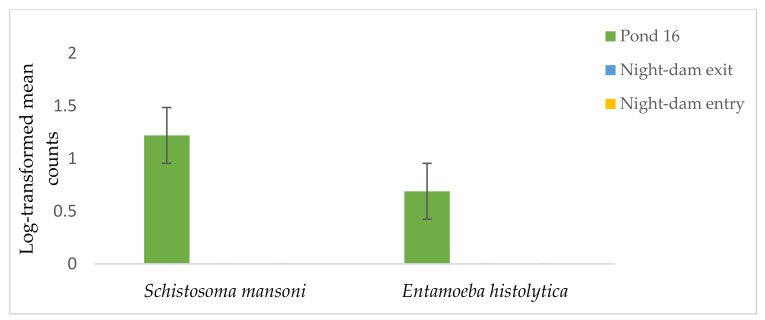
Distribution of log transformed Schistosoma mansoni and Entamoeba histolytica ova in different treated wastewater sources and borehole water used for irrigation at UL Experimental Farm.

Relative to Pond 16 in July, the night-dam entry increased *A. lumbricoides* by 22, 20, 12, and 35% in July, August, September, and November, respectively, however, the variable decreased by 28% in October (Table 3, Figure 6). Relative to Pond 16 in July, the night-dam exit decreased *A. lumbricoides* by 5 and 10% in July and August, respectively, however the variable decreased by 5, 9, and 12% in September, October, and November, respectively. There was no presence of *S. mansoni* or *E. histolytica* in the night-dam exit and night-dam entry (Figure 6). Therefore, relative to Pond 16, night-dam entry and night-dam exit decreased both variables by 100% (Table 4).

## 4. Discussion

### 4.1. Bacterial Counts

**Figure 7:**
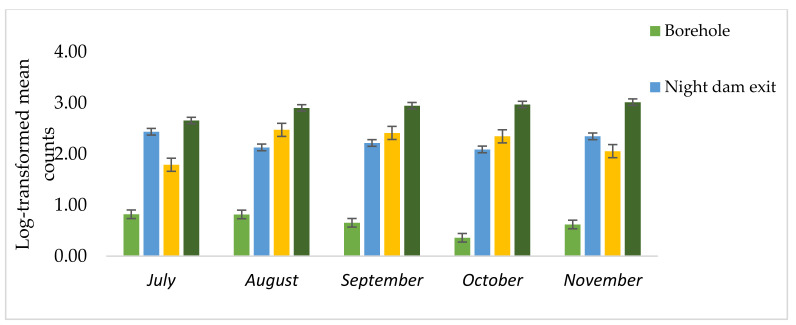
Log transformed salmonella sppcounts as distributed along the wastewater treatment pathway from Pond 16 exit to night-dam exit for five months in 2019.

The collection site–time interaction effect was significant for *Salmonella* spp., with negligent effects. However, the interactive effects were characterized by decreases and increases in counts at different sites and also exhibited changes with the seasons. Cooler months decreased the counts in the borehole location, whereas treated wastewater sources had increased counts (Figure 7). Available studies on interaction of post treatment storage and time effects on pathogenic contamination had varying results [21,22]. Fluctuating results were observed in *Salmonella* spp. counts for three years, which were higher in one summer and one winter, but low in one summer. Palacio et al. [22] reported a similar effect in storage tanks without assessing the effects of time on *Salmonella* spp. The presence of *Salmonella* spp. in borehole water was also supported by a number of studies that reported on the stability of the pathogen, which can survive up to 400 days when conducive temperatures prevail [23]. The decrease with time could be as a result of the organism’s survival abilities as *Salmonella* spp. could be affected predation by other organisms, among other things [22].

*Salmonella* spp. were the highest in the night-dam-entry when compared to the other sampling sites of treated wastewater (Figure 4). The decrease in *Salmonella* spp. in the night-dam exit could be due to the settling materials in the dam, as *Salmonella spp.* could be minimized by settling and exposure to heat [24]. The reported highest *Salmonella* spp. counts were in the same range with the set limit of 1000 CFU/100 mL [25]. The observed results were in agreement with the findings by a number of other studies [26,27]. Apparently, the use of this water could be unsafe for irrigation because *Salmonella* is a causal agent of gastroenteritis worldwide, with symptoms of infection including fever, nausea, and sometimes vomiting [28]. Therefore, irrigation water that is *Salmonella* positive could cause a health scare by contaminating vegetable produce, more especially those eaten raw [29].

The site–time interaction was not significant for *E. coli*, with the sampling site being highly significant for the counts of the pathogen. Contradictory findings have been reported on the effects of time on microbial makeup in irrigation water. *Escherichia coli* was reported to be higher in February and May than in September in a biofiltering study in Canada [22]. Another study at the stream networks of the Satilla River Basin, South Georgia, USA, reported higher *E. coli* counts in summer months than in winter [21].

*Escherichia coli* in the current study was present in all sampling sites, with low levels of contamination being in borehole water (Figure 4). However, in the night-dam exit, the counts were above 1000 CFU/100 mL, which showed an increase with movement and storage of treated wastewater post treatment. The findings were in a similar range to those observed by Al Amimi et al. [30]. Standards for reclaimed water use in agriculture had been set from the minimum counts of 5 to 300 CFU/100 mL for various vegetables [31,32]. Therefore, *E. coli* counts observed in the current study were higher than the recommended standards. *Escherichia coli* is the cause of illnesses such as gastroenteritis, which is inflammation of the gastrointestinal tract that involves the stomach and small intestine [33].

Fecal coliforms are the indicator bacteria most commonly used in discussions about wastewater reuse. The World Health Organization [25] set a limit of less than 1000 CFU/ 100 mL of Fecal coliforms, which is considered safe for wastewater use in irrigation. The reported highest count in the current study, which was observed in Pond 16, was higher than the set standards, but was lower than 1600 CFU/100 mL, which was detected elsewhere by others [34]. The absence of these bacteria in the borehole water was due to the fact that fecal coliform is associated with the presence of fecal materials from humans and other animals [35].

The site–time interaction had no significant effects on *Shigella* spp., with sampling time being highly significant for the variable. A contrasting study revealed the effects of sampling time on *Shigella* spp. in the North West Province of South Africa, where winter counts (176 CFU/100 mL) were reported to be higher than the summer counts (49 CFU/100 mL) [36]. The highest *Shigella* spp. counts were observed in the night-dam entry and Pond 16, with counts more than 500 CFU/100 mL. Borehole water exhibited the lowest counts of *Shigella* spp., followed by the night-dam exit. The results are in agreement with findings by Al Mimi et al. [30], who reported on 50 CFU/100 mL in a study where bacteriological quality of reclaimed wastewater used for irrigation was characterized in Dubai and Sharjah. In the present study, the low counts of *Shigella* spp. in the borehole samples could be due to exposure to unfavorable conditions including high soil pH, as *Shigella* spp. are regarded as fragile organisms that do not survive well outside their natural habitat [37] and are relatively heat sensitive organisms [38].

All wastewater samples and borehole water tested *Vibrio* spp. positive, although there were no time effects. The present findings are in contrast with the documented prevalence of *V. fluvaris*, as the organism is known to multiply in summer with the rise in water temperature [39]. Furthermore, *V. parahaemolytica* is associated with summer infections, indicating the prevalence of the organism in warmer temperatures [40]. The present findings concur with the results of a study that reported no seasonal effects for the explored prevalence of *Vibrio* spp. in final effluents from wastewater facilities in the Eastern Cape, South Africa [41]. However, other studies reported on a decrease in *Vibrio* spp. in winter when compared to summer [42].

The highest counts of *V. aginolytica* were observed in the night-dam exit (Figure 5). Whereas the highest counts of *V. cholera* of 890 CFU/100 mL as observed in the night-dam exit was above the WHO standard of 800 CFU/100 mL [25]. *Vibrio cholera* decreased with movement and storage of treated wastewater, as the lowest counts were observed in the night-dam exit. However, the presence of *V. cholera* in irrigation would still be harmful as it might lead to its presence on produce and this could cause cholera, which is an acute diarrheal infection [43]. The four species *V. fluvaris*, *V. parahaemolyticus*, *V. cholera*, and *V. aginolytica* each cause diarrhea, but in entirely different ways. *Vibrio parahaemolyticus* is an invasive organism, affecting primarily the colon, whereas *V. cholerae* is non-invasive, affecting the small intestine through secretion of an enterotoxin [44].

### 4.2. Helminths and Protozoa Counts

The site–time interaction was significant on *A. lumbricoides.* In Pond 16, the highest counts were observed during September, while night-dam entry and exit each had the highest counts in November. Studies on sampling site and time effects are not well-documented, with contradicting findings being available [45,46]. Amoah et al. [45] reported high *A. lumbricoides* in April–October, whereas November to March exhibited low counts. Additionally, *A. lumbricoides* counts were higher in the night-dam entry and Pond 16 during different months of sampling, which suggested that the pathogen decreased with storage after treatment. The increase with time towards summer months suggested that *A. lumbricoides* reproduced better in warmer temperatures. The observed number of ova concurred with other studies that have reported on the frequency of *A. lumbricoides* in raw and treated wastewater samples [47,48]. The observed counts in the current study were higher than the set standards of 1 ova/1 L water [49]. *Ascaris* is one of the most resilient of the enteric pathogens due to its resistance to external conditions [50], with the ova remaining viable for long periods, which serves as an indicator of the presence of the pathogen [51]. The presence of *A. lumbricoides* ova in treated wastewater in the current study could lead to contamination of irrigated vegetables and eventually lead to ascariasis in consumers. Globally studies have demonstrated the presence of *A. lumbricoides* in vegetables irrigated with treated wastewater [45,47,52].

The site–time interaction was not significant on *S. mansoni*. However, the sampling site was highly significant on the variable. The counts of *S. mansoni* and ova were not detected in borehole water, night-dam entry, or night-dam exit, but only occurred in Pond 16 exit. Formulated guidelines for the use of wastewater in unrestricted agriculture are available [25] with a value of less than 1 ova/L water being aimed for to reduce the risk of infection to consumers. Therefore, the counts of more than 10 ova/5 L water of *S. mansoni* in Pond 16 exit were higher than the standard and could be a threat to consumers and farm laborers. Recommendations by the World Health Organization [25] suggest that for most crop irrigation, the limit should be reduced to ≤0.1 ova/5 L water. Similarly, *E. histolytica* ova were only detected in Pond 16 exit and were not present at other sites. Worldwide, *E. histolytica* is a human intestinal protozoa associated with contaminated water and food [53]. The parasite is responsible for Amoebiasis, with symptoms including bloody diarrhea [54]. Therefore, the presence of this pathogen in irrigation water could lead to mortality for consumers and laborers. Observations at Pond 16 exit should not be viewed in isolation since it is not the point where water gets released for irrigation. The absence of *S. mansoni* and *E. histolytica* in the night-dam entry and exit could be explained by the short-term survival potential of the pathogen in exposed environments [55]. Additionally, the absence of both pathogens in other wastewater sampling points could be due to sufficient settling of the pathogens in the night-dam.

## 5. Conclusions

The results of the study demonstrated that all studied pathogens were significantly higher in treated wastewater samples when compared to the borehole samples. Additionally, for all microbial variables, counts were higher than the WHO standards. Generally, the microbial quality of treated wastewater in this study improved in the night-dam due to the afforded settling time. Usually, with proper treatment of the pathogens, the treated wastewater could ameliorate water scarcity pressures in semiarid regions with repeated drought incidents. In the current study, it would be prudent to have a chlorine station at the exit of the night-dam to further treat the water prior to release at the irrigation sites, with regular sampling and monitoring for pathogenic microbes being carried out prior to discharging the treated wastewater to the irrigated field in order to safe-guide the interest of workers and consumers. Additionally, the produce should also be tested for the presence of the test microbes. Moreover, it is recommended that a related study be carried out for 12 to 24 months to monitor the microbial loads over a longer period.

## Figures and Tables

**Table 1:** Log-transformed mean counts of *Salmonella* spp., *Shigella* spp., *Escherichia coli*, and fecal coliform in treated wastewater relative to that from borehole water used for irrigation of various crops.

Collection Site	*Salmonella* spp.		Fecal Coliform		*E. coli*		*Shigella* spp.	
	Variable ^y^	R.I. (%) ^z^	Variable	R.I. (%)	Variable	R.I. (%)	Variable	R.I. (%)
Borehole	0.65 ^c^	‒	0.00 ^d^	‒	1.45 ^c^	‒	1.63 ^b^	‒
Night-dam exit	2.24 ^b^	243	2.01 ^b^	‒	2.73 ^b^	88	1.87 ^b^	15
Night-dam entry	2.22 ^b^	239	1.78 ^c^	‒	2.86 ^ab^	97	2.69 ^a^	65
Pond 16 exit	2.90 ^a^	343	2.92 ^a^	‒	3.00 ^a^	106	2.67 ^a^	64

^y^ Column means followed by the same letter were not different (*p* ≤ 0.05) according to Duncan multiple range test. ^z^ Relative impact (%) = R.I. (%) = [(Wastewater/Borehole) − 1] × 100.

**Table 2:** Log-transformed mean counts of *Vibrio fluvaris* (*ViFlu)*, *Vibrio parahaemolytica (ViFlu)*, *Vibrio cholera (ViCho)*, and *Vibrio aginolytica (ViAgi)* as affected by collection site along the wastewater treatment pathway from Pond 16 exit to night-dam exit relative to those in the borehole water.

Collection Site	*ViFlu*			*ViPar*			*ViCho*			*ViAgi*		
	Untrans	Trans ^y^	R.l.(%) ^z^	Untrans	Trans	R.I. (%)	Untrans	Trans	R.l. (%)	Untrans	Trans	R.l. (%)
Borehole	2	0.42 ^a^	‒	10	1.01 ^d^	‒	22	1.06 ^d^	‒	0	0.00 ^d^	‒
Pond 16 exit	1	0.20 ^b^	−51	533	2.72 ^c^	169	741	2.85 ^a^	169	995	2.97 ^a^	‒
Night-dam entry	1	0.18 ^b^	−58	714	2.83 ^b^	180	494	2.68 ^b^	153	751	2.86 ^b^	‒
Night-dam exit	2	0.34 ^ab^	−19	896	2.94 ^a^	191	324	2.52 ^c^	138	317	2.50 ^c^	‒

^z^ Relative impact (%) = R.I. (%) = [(Wastewater/Borehole) − 1] × 100. Untrans = Untransformed counts; Trans = transformed counts; ^y^ Column means followed by the same letter were not different (*p* ≤ 0.05) according to Duncan multiple range test.

**Table 3:** Log transformed *Ascaris lumbricoides* counts as distributed along the wastewater treatment pathway from Pond 16 exit to night-dam exit for five months in 2019.

Collection Site	July		August		September		October		November	
	Variable ^y^	R.I. (%) ^z^	Variable	R.I. (%)	Variable	R.I. (%)	Variable	R.I. (%)	Variable	R.I. (%)
Pond 16 exit	1.10 ^ab^	−	1.22 ^ab^	11	1.26 ^ab^	14	1.15 ^ab^	4	1.13 ^ab^	3
Night-dam entry	1.34 ^a^	22	1.32 ^a^	20	1.23 ^ab^	12	0.79 ^b^	−28	1.48 ^a^	35
Night-dam exit	1.04 ^ab^	−5	0.99 ^ab^	−10	1.16 ^ab^	5	1.20 ^ab^	9	1.23 ^ab^	12

^y^ Column means followed by the same letter were not different (*p* ≤ 0.05) according to Fisher’s least significant test. ^z^ Relative impact (%) = R.I. (%) = [(Wastewater/Borehole) − 1] × 100.

**Table 4:** Distribution of log transformed *Schistosoma mansoni* and *Entamoeba histolytica* ova in different treated wastewater sources and borehole water used for irrigation at UL Experimental Farm.

Collection Site	*S. mansoni* ^y^	R.I. % ^z^	*E. histolytica*	R.I. %
Pond 16	1.22 ^a^	−	0.69 ^a^	−
Night-dam exit	0 ^b^	−100	0 ^b^	−100
Night-dam entry	0 ^b^	−100	0 ^b^	−100

^y^ Column means followed by the same letter were not different (*p* ≤ 0.05) according to Fisher’s least significant test. ^z^ Relative impact (%) = R.I. (%) = [(Wastewater/Borehole) − 1] × 100.

## Data Availability

The data presented in this study are available in a separate document.
